# Development of pseudo-subarachnoid hemorrhage secondary to hypoxic-ischemic injury due to bleeding pulmonary arterio-venous malformation

**DOI:** 10.1186/s12883-018-1161-x

**Published:** 2018-09-28

**Authors:** Mohamad Syafeeq Faeez Md Noh, Anna Misyail Abdul Rashid

**Affiliations:** 1 Department of Imaging, Faculty of Medicine and Health Sciences, Universiti Putra Malaysia, Seri Kembangan, Malaysia; 2 Department of Medicine, Faculty of Medicine and Health Sciences, Universiti Putra Malaysia, Seri Kembangan, Malaysia

**Keywords:** Pseudo-subarachnoid hemorrhage (SAH), Pulmonary arterio-venous malformation (AVM), Computed tomography (CT)

## Abstract

**Background:**

The computed tomography (CT) finding of a pseudo-subarachnoid hemorrhage (SAH) may lead the treating physician into a diagnostic dilemma. We present a case of a pseudo-SAH in a patient with post-resuscitative encephalopathy, secondary to a newly diagnosed bleeding pulmonary arterio-venous malformation (AVM).

**Case presentation:**

A 19-year-old female presented acutely with massive hemoptysis. Cardiopulmonary resuscitation (CPR) followed, and the patient was subsequently intubated for airway protection with intensive care unit (ICU) admission. Urgent CT angiography of the thorax showed a bleeding pulmonary AVM, with evidence of hemothorax. Non-contrasted cranial CT initially revealed cerebral edema. Day 3 post admission, repeat cranial CT showed worsening cerebral edema, with evidence of pseudo-SAH. Patient passed away the next day.

**Conclusions:**

Pseudo-SAH, if present, carries a poor prognosis. It should be recognized as a potential CT finding in patients with severe cerebral edema, due to various causes. The diagnosis is vital, to avoid wrongful treatment institution, as well as determination of cause of death.

## Background

Acute SAH is seen on cranial CT as increased density of the basal cisterns and subarachnoid spaces [1]; the finding of which, in the absence of trauma, may necessitate further tests to determine the cause. However, a false positive diagnosis of an acute SAH may bring about potential problems – wrongful treatment, unnecessary workup for genetic predispositions, and difficulties in determining cause of death. A potential mimic to a true acute SAH, among others, is an entity called pseudo-SAH. This condition usually arises in the setting of a hypoxic encephalopathy, due to various causes. A combination of cerebral edema (with resultant reduced attenuation of the brain parenchyma), effacement of the subarachnoid spaces, as well as engorgement of venous structures in the pial surfaces leads to its perceptual high attenuation on cranial CT – leading to a false diagnosis of an acute SAH [2]. We report, in our experience, a young female who initially presented with massive hemoptysis, whom on serial cranial CT had findings of a pseudo-SAH.

## Case presentation

**Fig. 1:**
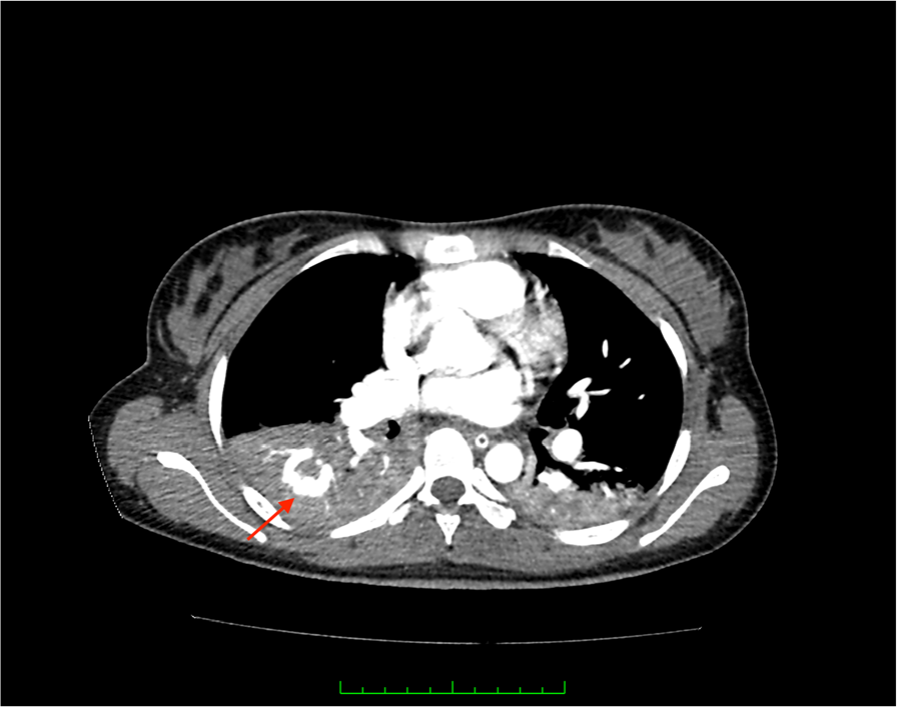
CT angiography of the thorax, in axial section showing presence of a pulmonary AVM (red arrow). Note also presence of hemothorax.

**Fig. 2:**
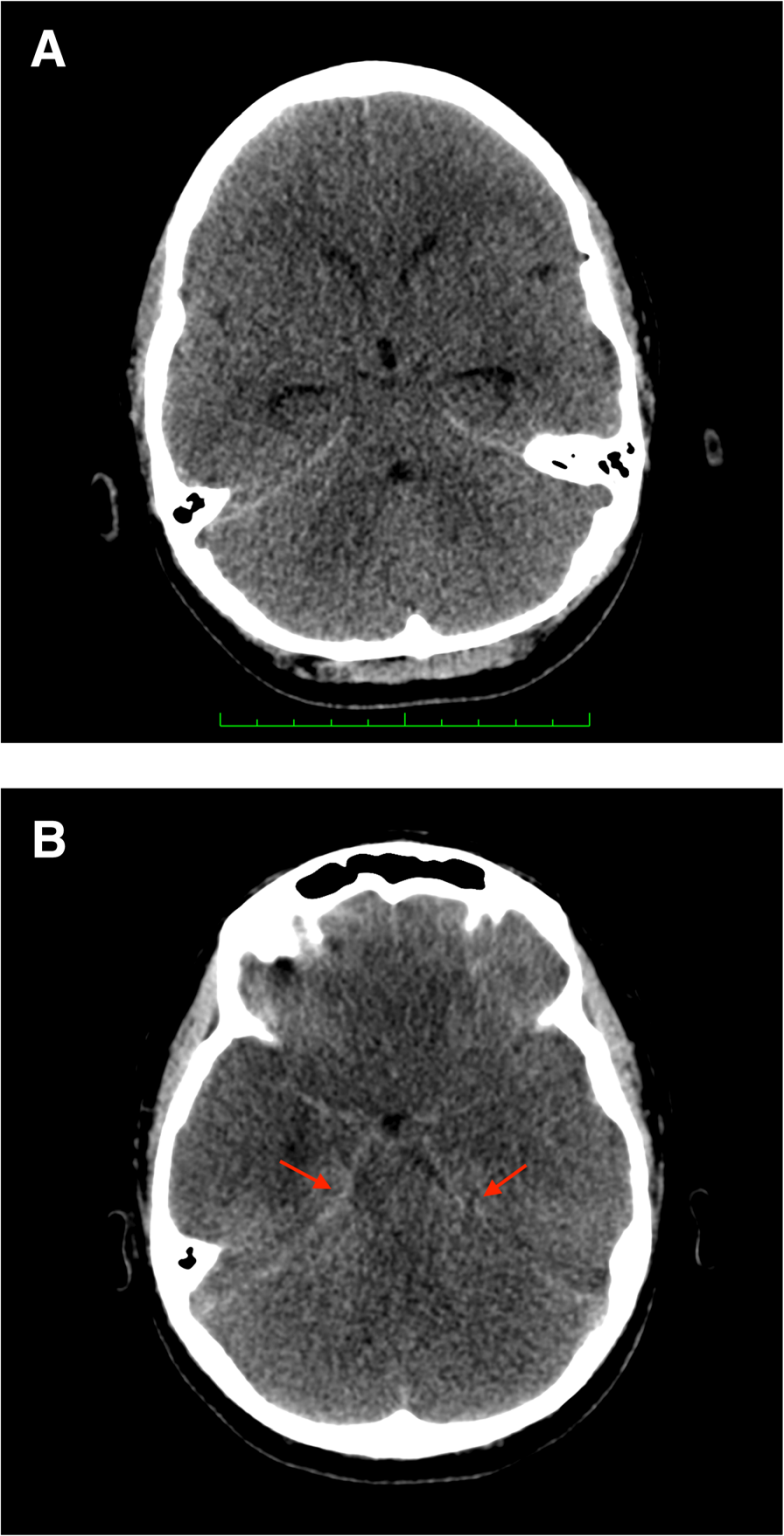
**a** Non-contrasted cranial CT, on day of admission, taken at the level of the basal cisterns, in axial section showing evidence of cerebral edema, with mild loss of grey-white matter differentiation. **b** Non-contrasted cranial CT, on day 3 of admission, taken at the level of the basal cisterns, showing evidence of high density areas (red arrows) indicative of pseudo-SAH. Note that the cerebral edema is worse compared to the initial CT (**a**)

A 19-year-old female, with no underlying medical illness presented acutely to the Emergency Department with massive hemoptysis. Upon arrival, she was noted to be in asystole. Pupils were 4 mm bilaterally, non-reactive. CPR was commenced, and continued for 20 min until she was revived. Urgent blood work revealed a hemoglobin of 2.3 g/dL (normal range 12–15 g/dL), normal coagulation profile, and arterial blood gases indicative of metabolic acidosis. She was intubated for airway protection, and transferred to the ICU for further management. An urgent CT angiography of the thorax showed a right sided pulmonary AVM, with evidence of active bleeding (hemothorax) (Fig. 1). Non-contrasted cranial CT revealed cerebral edema (Fig. 2a). At this juncture, a decision was made to embolize the bleeding pulmonary AVM, should cerebral resuscitation show improvement. On day 3 of admission, repeat cranial CT showed dense basal cisterns and subarachnoid spaces (Fig. 2b), with marked worsening of the initially seen cerebral edema. A neurological consult was sought at this point, to assess the brain function, anticipating a possibility of brain death. The brain stem reflexes were absent, compatible with brain death. Additionally, the deep tendon reflexes were depressed, and the Babinski’s response was up-going. An electroencephalography was not pursued. Taking into account the previous history of resuscitation, worsening cerebral edema with a clinical diagnosis of brain death, stable hemoglobin level post transfusion, as well as fixed and dilated pupils (7 mm bilaterally), this is recognized to be a pseudo-SAH. Combined with the worsening cerebral edema and loss of grey-white matter differentiation, findings are suggestive of hypoxic-ischemic injury. The family was counselled, and decided to discontinue life support. Patient subsequently passed away.

## Discussion and conclusions

The radiological sign of pseudo-SAH was initially described in 1986, by Spiegel et al. [3]. At the beginning, diffuse cerebral edema was the attributed cause of this finding. Avrahami et al. [4] later reported that contrary to the previous understanding that this is a rare phenomenon, it is in fact common; detected in younger patients (below 40 years old) with high cerebral/skull volume, usually in the context of drug abuse, trauma, or cardiorespiratory arrest, leading to cerebral edema as a result of the hypoxic-ischemic injury. At present, multiple reports and studies have documented other causes of this finding, which include meningitis, subdural hemorrhage, infarction, contrast administration, spontaneous intracranial hypotension, post myelography, polycythemia, and chronic hypoxaemia, among others [5–7].

The dilemma between diagnosing a pseudo-SAH and a true acute SAH lies most importantly in the knowledge of the existence of such a phenomenon. Opeskin and Bedford et al. [8, 9], in their experience, encountered cases where antemortem acute SAH was diagnosed, leading to autopsy for confirmation. Upon reviewing the cranial CT scans available, as well as post mortem examination, there was no evidence of hemorrhage in the subarachnoid spaces. This highlights the importance of knowing the possible etiological factors of a pseudo-SAH and recognizing it when encountered. This avoids unnecessary post mortem examinations and wrongful determination of cause of death in some cases. Additionally, since one of the causes of a non-traumatic subarachnoid hemorrhage is a Berry aneurysm, which is potentially inherited, this avoids unnecessary screening of family members.

Yuzawa et al. [10] in 2008 showed that pseudo-SAH can be diagnosed using the CT parameters available at the physician’s disposal. In their study, they compared those with a pseudo-SAH, against those without. The Hounsfield unit (HU) of the high density areas were measured and compared. In those with pseudo-SAH, the HU values at the high density areas were in the range of 30–42 HU, with slight fluctuation with time. This is in contrast with those with a true SAH; where the values were higher (and decreased with time). Our patient had a HU value between 40 and 42 at the basal cisterns, corroborating this finding. In addition, they also looked at the onset of pseudo-SAH in their series. On day 3 of imaging, all of their patients exhibited CT findings of pseudo-SAH. This was also seen in our experience.

The underlying mechanism for the development of a pseudo-SAH till this day remains uncertain, although many postulations have been put forward [4, 5, 10, 11]. Among these, the most mentioned pathogenetic mechanism is the compression of the dural sinuses, leading to compromise of venous flow resulting in superficial veins engorgement – which appears as high dense areas in the background of low attenuated, edematous brain matter. Despite being engorged, this venous blood is still within the vasculature. Blood leaking from the vasculature has higher attenuation values due to the rapid plasma absorption, thus strengthening the study findings by Yuzawa et al. [10], that lower attenuation values seen in the high density areas suggest a pseudo-SAH, rather than a true SAH.

In conclusion, pseudo-SAH, despite being an entity that till this day remains unknown in frequency, should be recognized when encountered by treating physicians. Knowledge of the risk factors, pathogenetic mechanisms, and appearance on CT enables accurate and timely diagnosis – avoiding unnecessary tests and procedures.

## Abbreviations

Pseudo-SAH: Pseudo-subarachnoid hemorrhage
Pulmonary AVM: pulmonary arterio-venous malformation
CT: Computed tomography
CPR: Cardiopulmonary resuscitation
ICU: Intensive care unit
HU: Hounsfield unit
